# Supplementary material for: Insight into Carbohydrate Metabolism and Signaling in Grapevine Buds during Dormancy Progression
Source: Plants (Basel). 2022 Apr 9;11(8):1027. doi: 10.3390/plants11081027 (PMC9028844; doi:10.3390/plants11081027)
Supplement: Supplementary file 1 [file plants-11-01027-s001.zip › plants-1653275-supplementary.pdf]

Table S1. List of primers used for qPCR analysis. *VvMSA* = Maturation, Stress, ABA; *VvHT1* = Hexose Transporter 1; *VvHT5* = Hexose Transporter 5; *VvRS* = Raffinose Synthase.

Gene target		Primer sequence (5′-3′)	Accession
VvHT5[7]	F	CTTTCCATGCTTTGCCATTT	Vitvi05g00468
	R	ACCAATGCTTCTTCCACACC	
VvHT1[35]	F	TCAACGATGGTTCTTACAGC	Vitvi10g00358
	R	AACCGATAGTATTGTATTCG	
VvINV[42]	F	GCAGCAGAAATGGGGTTGAA	Vitvi04g00094
	R	AGGTATCGGTTTCAGGCACA	
VvMSA[43]	F	GCATGTGTGCTTGTTGTGTAA	Vitvi18g02973
	R	TCACAAGGACACACAGAGAGA	
VvRS	F	CTCTCCCGGGGAAATCTGTT	Vitvi14g01717
	R	GATCTTGGTTTCTCGGCTGC	

Table S2. Results of statistical analysis performed separately for each sugar.

	Glucose	Fructose	Sucrose	Raffinose
Oct 22nd	*c*	*d*	*b*	*bcd*
Nov 11th	*c*	*cd*	*b*	*ab*
Nov 25th	*c*	*d*	*b*	*bcd*
Dec 15th	*a*	*a*	*a*	*a*
Dec 29th	*ab*	*ab*	*ab*	*abc*
Jan 9th	*bc*	*abc*	*b*	*d*
Jan 23rd	*c*	*bcd*	*b*	*d*
Feb 11th	*bc*	*bc*	*ab*	*bcd*
Feb 25th	*c*	*bcd*	*b*	*cd*
Mar 10th	*c*	*bcd*	*ab*	*cd*
